# Action 3:30: protocol for a randomized feasibility trial of a teaching assistant led extracurricular physical activity intervention

**DOI:** 10.1186/1745-6215-14-122

**Published:** 2013-05-02

**Authors:** Russell Jago, Mark J Edwards, Ashley R Cooper, Kenneth R Fox, Jane Powell, Simon J Sebire, Melissa Spears, Janice L Thompson, Alan A Montgomery

**Affiliations:** 1 Centre for Exercise, Nutrition & Health Sciences, School for Policy Studies, University of Bristol, Bristol, UK; 2 Faculty of Health and Life Sciences, University of the West of England, Bristol, UK; 3 MRC Clinical Trials Unit, London, UK; 4 School of Sport and Exercise Sciences, University of Birmingham, Birmingham, UK; 5 Nottingham Clinical Trials Unit, University of Nottingham, Nottingham, UK

**Keywords:** Children, Feasibility trial, Intervention, Physical activity, Teaching assistant

## Abstract

**Background:**

Many children do not meet physical activity (PA) guidelines. Extracurricular programmes could provide a mechanism to increase the PA levels of primary-school-aged children. Teaching assistants (TAs) are a valuable resource in all UK primary schools and could be trained to delivery after-school PA programmes. The aim of this feasibility study is to examine whether the Action 3:30 PA intervention, which is delivered by TAs, could be effective in increasing the PA of Year 5 and 6 children.

**Methods/Design:**

A feasibility trial will be conducted in 20 primary schools. Schools will be randomly assigned to intervention or control arms. Intervention schools will receive a 25-hour TA training programme for two TAs, a first-aid certificate course for two TAs; ongoing TA support; 40 one-hour session plans that can be delivered by TAs; Action 3:30 clubs that run twice a week for 20 weeks; and ten sets of parent information sheets that are distributed biweekly.

All measures will be assessed at baseline (Time 0), at the end of the intervention period (Time 1) and four months after the intervention has ended (Time 2). As this is a feasibility study, our primary interest is in estimating the recruitment of schools and children, adherence to the intervention, and completeness of data collection for outcomes and costs.

As the most likely primary outcome measure in a future definitive trial will be accelerometer-determined minutes of moderate-to-vigorous PA (MVPA) per day, participants will wear accelerometers for five days (including two weekend days). Several psychosocial variables that could act as mediators in a future trial will be assessed via a questionnaire. Process evaluations of the session attendance, perceived enjoyment and perceived exertion will be assessed during the intervention. At the end of the intervention period, qualitative assessments will be conducted to identify how the programme could be improved before proceeding to a larger trial.

**Discussion:**

The goal of the feasibility trial is to assess the potential of this innovative intervention approach and provide all the information necessary to design a cluster randomized controlled trial.

**Trial registration:**

ISRCTN, ISRCTN58502739

## Background

Physical activity (PA) is associated with reduced body mass, healthier blood lipid profiles, lower blood pressure, lower insulin levels and enhanced mental well-being among children [1]. Despite the benefits of regular PA, many young people do not meet the current UK recommendation of an hour of PA on most days of the week [2]. The end of primary school (UK school years 5 and 6, approximate age, 9 to 11 years) is a period when children’s PA levels start to decline [3,4].

Opportunities for children to be active can occur both within and outside the school curriculum. Physical activity during the primary school curriculum in the UK is often limited to two hours of physical education (PE) per week [5]. As a result, the school curriculum rarely provides enough opportunities for children to meet PA guidelines or adequately develop their physical skills. One solution is to develop noncurriculum-based activities to be delivered immediately after the formal school day. This period may be regarded as children’s discretionary PA time and children who are inactive after school are less likely to meet PA guidelines [6]. As such, interventions delivered in the after-school period could become a means of increasing PA among primary school-aged children.

After-school clubs are a central element of the UK Government’s ‘Extended schools’ strategy for primary schools [7] and many children participate in supervized programmes for additional academic support, music, creative activities and competitive sports. Organized after-school PA programmes that focus on increasing PA opportunities for a wide group of children could therefore be an effective means of engaging inactive children in PA [8]. A recent systematic review reported five evaluations of after-school PA interventions that had employed objective evaluation methods [8]. Of the five studies, three interventions reported positive effects on PA while a fourth pilot study reported a trend towards increased PA when compared with a control group. Four of the five interventions were well-received by the children and their parents. With the exception of one study conducted in Spain [9], all studies were conducted in the USA. Thus, although some UK schools offer organized after-school PA programmes, a rigorous and systematic evaluation of this type of intervention has not been conducted.

Teaching assistants (TAs) are support staff who usually work in a classroom setting by assisting the teacher and supporting pupils. As there are TAs in all UK primary schools they form a unique human resource and could be trained to deliver after-school PA programmes. Delivering after-school PA via TAs is advantageous because it is consistent with the UK Extended Schools [7] and National Institute for Health and Clinical Excellence (NICE) [10] guidance; commercially based activity session providers are expensive; and it allows schools to develop the skills of their staff. An after-school PA intervention delivered by TAs could therefore be a cost-effective and scalable intervention for UK primary schools.

Behaviour-change interventions that have been based on psychological theory have been more successful than those that have not, and can provide key advances for intervention design, as they facilitate the identification of key mediators of behaviour change [11]. Self-determination theory [12] may be particularly appropriate for understanding children’s PA levels. Self-determination theory focuses on motivations for behaviour and contends that being motivated for autonomous or intrinsic reasons (that is, because PA is fun or provides valued benefits, such as feelings of competence or spending time with friends) leads to more positive cognitive, affective and behavioural outcomes than does being motivated by externally controlled reasons. Evidence from the PE and psychology literature indicates that autonomous motivation is associated with positive outcomes in children and adolescents, such as self-reported exercise behaviour, pedometer counts, quality of life and positive self-concept [13,14]. Autonomous motivation and psychological well-being are facilitated when three innate needs are satisfied: autonomy (acting with choice and self-endorsement); competence (feeling effective in one’s environment); and relatedness (feeling a mutual sense of connectedness with others [12]. Thus, programmes that help primary school children feel more physically competent and confident of being active, that show that their efforts are key to their success, and that engender fun and being part of a supportive team, are likely to optimize children’s motivation to engage with the intervention and stay involved in PA.

Self-determination theory suggests that motivation is influenced by the social environment created by significant others [15]. For example, teachers can enhance autonomous motivation by being autonomy-supportive (engaging children in decision-making), by providing structure (clear expectations and guidelines) and by being interpersonally involved (for example, showing empathy with pupils). Leaders who use these empowering strategies have positive effects on pupils’ motivation, behavioural engagement and psychological well-being [15]. Thus, TAs could be trained in providing developmentally appropriate curricular content using a delivery style that fosters optimal motivation and development. To achieve this objective, TAs will also need training in the safe management of the PA environment and the activities within each session using a style that fosters enjoyment, enthusiasm and autonomy [16].

In light of this background evidence, we hypothesized that TA-led after-school programmes could be a means of promoting PA among primary school children. To test this hypothesis, a feasibility trial evaluation of a new intervention, Action 3:30, will be conducted. The main research question for a future definitive trial is, ‘Is Action 3:30, an after-school PA intervention that is based on behaviour-change theory and delivered by TAs, effective in improving the PA, attitudes and confidence of Year 5 and 6 children?’ The specific aims of the present feasibility study are to:

1. Estimate the likely recruitment, attendance and retention rates of TAs and pupils to the Action 3:30 after-school PA intervention.

2. Estimate the variability of PA and secondary outcomes at baseline and 20-week follow-up.

3. Estimate outcome intraclass correlation coefficients.

4. Estimate the outcome completion rate (provision of data) four months after the intervention ends and identify any potential differences in completion between those who remain in primary school (Year 5) and those who move to secondary school (Year 6).

5. Develop a framework to facilitate conducting a cost-effectiveness analysis of the intervention.

6. Estimate the sample size for an adequately powered evaluation of the Action 3:30 intervention.

7. Conduct post-intervention qualitative research with key stakeholders to refine the design and delivery of Action 3:30.

## Methods/Design

Action 3:30 is an after-school PA programme that is taught by TAs within the school (see intervention details). The feasibility trial will be conducted in 20 primary schools recruited from the Greater Bristol area. Randomization will take place once all schools have been recruited and after baseline data collection. Balance between trial arms will be achieved with respect to local education authority membership, deprivation, ratio of Year 5 to Year 6 pupils, proportion of female participants, and mean minutes of moderate-to-vigorous PA (MVPA) at baseline. This will be achieved by calculating an imbalance statistic [17] for all possible allocation sequences with at least two intervention schools per local education authority, then randomly selecting one sequence from a subset with the most desirable balance properties. This process will be performed by an independent statistician.

We aim to recruit 30 Year 5 or 6 pupils from each primary school. Previous research indicates that a majority of children are not currently meeting PA recommendations [3,4] and almost all children would benefit from an increase in PA. The programme will be oriented to general physical skills development and activity rather than specific sports skills, with the hope of attracting children not already involved in team sports. Therefore, all Year 5 and 6 children who are physically able to engage in PE classes will be eligible to participate in the sessions. In schools where more than 30 children consent, and are eligible to participate, 30 will be randomly selected for the study by the research team.

The project was approved by the ethics and research committee of the School for Policy Studies at the University of Bristol. Written informed parental consent was obtained for all participants.

### The Action 3:30 intervention

The Action 3:30 intervention is based on training TAs to deliver PA sessions after school and providing all of the resources and support that are necessary to run Action 3:30 clubs in the intervention schools. The six specific elements of the Action 3:30 intervention are summarized in Table 1.

**Table 1:** Action 3:30 intervention components

**Component**	**Description**
**25-hour TA training programme**	Community Sports Leader Award (tailored for teaching assistants)
	FUNdamentals (Bristol City Council in-house training programme that focuses on fundamental movement skills)
	Focused sessions on learning about motivation and delivery to pupils in an autonomy-supportive manner
**First-aid certificate course**	One-day first-aid training course
**Ongoing TA support**	One-day ‘booster’ training session for TAs, to occur after the first 10 weeks of the intervention
	One visit per school by trainer to support TAs during the delivery of the sessions
	Three hours of phone or email support per school from trainer
**Session plans**	40 one-hour session plans
**Action 3:30 clubs**	Trained TAs run a 1-hour Action 3:30 session at the school site twice a week for 20 weeks
	TAs deliver content on Action 3:30 session plans
**Parental information sheets**	Biweekly (every four sessions); outlines activities that have been taught during the previous two weeks
	Children and parents provided with ideas of activities that the children could engage with parents, siblings or friends to reinforce activity session materials

TA, teaching assistant.

#### TA training programme

Two TAs in each intervention school will attend a 25-hour educational programme that focuses on delivering PA sessions after school. The training programme is a modification of an existing local council programme. The content was refined in light of interviews that were conducted with TAs who had previously attended a similar Bristol City Council training programme. The interviews highlighted the most beneficial aspects of the training from the TAs’ perspective, for example, the importance of communication with children, adapting games and activities for pupils with different levels of ability, and having the opportunity to deliver practice sessions to children. Interview findings were shared with the training course leader to refine the programme content. The training will be led by the Coach Development Officer (CDO) for Bristol City Council. The programme will lead to the Community Sports Leader Award (CSLA), and will be specifically tailored for TAs delivering PA sessions to Year 5 and 6 children. The CSLA is an established award that also fulfils the requirements of a Level 2 award on the Qualifications and Credit Framework. During the training programme, the TAs will also follow the FUNdamentals training programme. This is a 3-hour Bristol City Council in-house course that focuses on teaching leaders to increase children’s fundamental movement skills, including jumping, hopping, skipping, running and stopping safely.

The 25-hour training programme includes a one-hour session in which the TAs are taught the fundamental principles of self-determination theory and practical applications for teaching and motivating young people. Throughout the 25-hour course, the TAs will be trained to use an autonomy-supportive style that acknowledges feelings and preferences, conveys a sense of choice, and provides support for children’s autonomy, competence and relatedness [18]. Activities that reinforce these concepts are included in each of the five days of the training programme. Moreover, to engender feelings of belonging and relatedness, sessions will feature cooperation, social interaction and a ‘club’ spirit. The 5-day training programme includes opportunities to practise delivering PA sessions to Year 5 and 6 students with feedback on delivery style and group management. To maximize the appeal of Action 3:30 and to reduce any financial burden schools may incur, schools will be reimbursed for TA time to attend the training programme. A more detailed overview of the structure of the training programme is presented in Table 2.

**Table 2:** Outline of training programme

**Session**	**Details of content**
Introduction: CSLA module 1 (all CSLA modules adapted for primary school teaching assistants’ needs)	General introduction to course
	Introduction to communication and motivation techniques
	Planning and organization of intervention session plans
CSLA modules 2 and 5	Adapted games (for 9–11 year old children)
	Warm-up skills, delivering safe sessions, and how to lead sessions
FUNdamentals: agility, balance and coordination	Fundamental movement skills. Basic skills that include walking, running, stopping, jumping, and catching safely
	How to develop and deliver mini games for a range of activities and sports
CSLA module 1 (continued), 3, 4, 5, 7	Understanding fitness and safety
	Participants design mock after-school session for children
	Assessment planning for Session 5
CSLA module 7: assessment	Participants to complete log books
	Deliver planned session (Session 4) to children in a local school
	Course evaluation and completion of paperwork
	Discussions of ‘what next’

TA, teaching assistant.

#### First-aid certificate

All TAs will need to attend a one-day first-aid course to fulfil the criteria of the CSLA award. This training will be provided for all staff who do not currently have this qualification.

#### Ongoing TA support

All TAs will be provided with ongoing support from the CDO for Bristol City Council during the intervention period. The CDO will visit each school once to observe an Action 3:30 club in progress. At the end of the observation period, the CDO will discuss with both TAs the ways in which their delivery could be improved. In addition, the CDO will provide three hours of telephone or email support per school (approximately 1.5 hours per TA in the school). The TAs will also be provided with a one-day booster session, delivered by the CDO, midway through the intervention period.

#### PA session plans

Intervention TAs will be provided with a project manual (*Action 3:30 Leaders’ Manual*) that includes 40 session plans (two per week for 20 weeks). To ensure that the activities are attractive to all children, regardless of ability, sex or sporting experience, the session plans use a range of adaptable activities to improve hand-eye and limb coordination and the agility skills that are the building blocks of many sports and activities. The foundation of each session will be enjoyable physical activities including games, pair work, and individual challenges.

#### Action 3:30 clubs

Action 3:30 clubs will be instigated in each of the ten intervention schools. The clubs will run for one hour after school, twice per week, for 20 weeks. During the clubs, the two TAs that have attended the training programme will deliver the session plans.

#### Parental information

Information sheets will be sent home with intervention participants every two weeks. Each information sheet will be a double-sided page that includes information on what was taught during the previous four Action 3:30 sessions and related practice activities and games that the child can engage in with parents, siblings or friends outside of the structured sessions. These materials are designed to ensure that parents are aware of the activities that are being taught during the sessions; and to encourage children to use the skills and knowledge learnt during the sessions to engage in additional activity at home.

### School and student appreciation

Intervention schools will receive TA training, funds to cover TA training and delivery time, and £200 of equipment to deliver the Action 3:30 programme. The TAs will be paid for two hours of additional time per week for the duration of the project and to attend project training. Control schools will receive a £200 donation to the school fund, a one-day training programme for two TAs in the school once the intervention has been completed, and a copy of the *Action 3:30 Leaders’ Manual*.

As we have found in previous research [19] that incentivizing data provision is necessary for intervention and control groups, incentives will be provided for all participants at each time point. Children will receive a small thank-you gift (worth £1) for each of the first two data collections. The gifts will be selected specifically to promote PA. This small gift is designed to ensure that all accelerometers are returned promptly, thereby facilitating the necessary rapid data collections at each phase of the study. To maximize return rates when approximately half of the sample will be in secondary school, we will provide a £10 thank-you gift for the final data collection.

### Measures

All measures will be assessed at baseline (Time 0), during the last few weeks of the intervention period (Time 1), and four months after the intervention has ended (Time 2). As this is a feasibility study, our primary interest is in estimating the recruitment of schools and children, adherence with the intervention, and completeness of data collection for outcomes and costs.

The most likely primary outcome measure in a future definitive trial will be accelerometer-determined MVPA per day. Accelerometers provide accurate and reliable assessments of PA among young people [20]. Participants will wear accelerometers for five days (including two weekend days). The accelerometers will be set to record at 10 second intervals. Periods greater than an hour with zero values will be considered nonwear time and will be removed from the data. Mean minutes of MVPA will be established for weekdays and weekend days using cut-points developed for children [21]. Accelerometer counts per minute, an indication of the volume of activity in which the children engage, will be also be derived. As the intervention is specifically focused on the after-school period, we will also assess both MVPA and counts per minute during the after-school period (3:30 pm to 8:30 pm). All accelerometry variables will be continuous.

It is envisaged that in a future trial, the primary comparison will be at Time 1, with this assessment designed to assess the effect of attending the Action 3:30 clubs while they are still running. The Time 2 assessments are a secondary comparison designed to establish if there is a longer-term effect once the intervention sessions have ended. (Participants who were in Year 6 at the baseline assessment (approximately 50%) will have transitioned to Year 7 in secondary schools at the Time 2 assessment.)

As it is possible that an increase in PA after school might reduce opportunities to engage in screen-viewing we will assess child self-reported screen-viewing at each of the three assessment periods. We will also assess five further self-reported (continuous) variables that were selected as they could be mediators of behaviour change in a full trial. The five constructs are:

1. Autonomous and controlled motivation for PA. This will be measured using items from the Behavioural Regulations for Exercise Questionnaire [22] adapted for this age group along with some newly developed items. Adaptations were made to simplify the language of some items while retaining the meaningful item content and to refer to PA rather than exercise.

2. Perceived level of satisfaction of PA-based autonomy, competence and relatedness needs [23,24].

3. Perceived enjoyment of PA [24].

4. Global self-esteem, measured using the ‘general’ sub-scale of the Self-Description Questionnaire-I [25].

5. Maternal and paternal PA support (logistic, modelling and sedentary restriction), measured using the Activity Support Scale [26,27].

Children’s height and weight will be assessed with their shoes, coats and jumpers removed. Height will be measured to the nearest 0.1 cm using a portable SECA stadiometer. Weight will be measured to the nearest 0.1 kg using a digital SECA scale. Body mass index (kg/m^2^) will be calculated and converted to an age- and sex-specific body mass index standard deviation score [28].

#### TA questionnaire

The TAs who deliver the intervention will be asked to report age, sex and education level at baseline. They will also be asked to self-report teaching efficacy [29] and provision of autonomy support [30] at four time points: on recruitment and prior to training; after training; at the mid-point of the intervention; and at the end of the intervention period.

#### Process evaluation

The number of sessions per week and the number of children attending each session will be recorded by the TAs in each of the ten intervention schools. All children in the intervention schools will be asked to complete perceived exertion [31] and perceived enjoyment of the Action 3:30 session [32] on ten occasions during the 20-week period, (once every two weeks). Children will also report their perceived autonomy support provided by the TAs on four occasions (every five weeks) using an adapted version of the Health Care Climate Questionnaire [33].

#### Economic assessment

One aim of the feasibility trial is to develop a framework to perform a cost-effectiveness evaluation of the intervention if a full trial evaluation is conducted in the future. As such, the feasibility trial will focus on identifying all of the inputs that would be necessary for such an evaluation at four stages. This approach to deriving costing follows the methods applied in recent research in a school setting [34]. Resources used at stage one (project development), stage two (planning), stage three (delivery) and stage four (present the Action 3:30 programme) will be identified separately. By collecting this cost information we will be able to assess the feasibility, appropriateness, advantages and disadvantages of the data collection methods and tools that would be needed for the economic evaluation of a trial. Timesheet and expense data will be collected for all resources used to deliver the Action 3:30 intervention at each stage of planning and delivery, from a public sector perspective.

#### Post-study qualitative work

Qualitative research will be conducted with intervention participants, TAs and school administrators at the end of the intervention to inform any necessary revisions to elements of the intervention training and delivery. The qualitative work that will be conducted with each group is summarized next.

##### Intervention participants (children)

A focus group will be conducted –in each intervention school. The groups will include high-attending pupils (pupils in the middle and highest thirds of attendance) and low-attending pupils (pupils in the lowest third of attendance). We aim to achieve a balance of boys and girls in each group. The focus groups will examine aspects of the programme that the children enjoyed, elements they did not enjoy, factors that either positively or negatively affected recruitment and any suggestions on how to improve the intervention.

##### Teaching assistants

Intervention school TAs will be asked to take part in a semi-structured interview focusing on their experiences of the Action 3:30 project, including their opinions of the training and intervention resources, session delivery, areas of success and challenge, and recommendations for refinement of the intervention. The TAs will also be asked to comment qualitatively on how the training programme affected their perceived ability to lead PA sessions and their thoughts on what would need to be done to maintain this provision once the intervention ends.

##### School administrators

We will interview the member of staff at five intervention and five control schools (not the TAs) who was the main project liaison or administrator contact. The schools will be purposefully selected based on the implementation rates (high or low) of the project. The role of this person will vary between schools but it is likely that she or he will be either head or deputy head teacher, a head of Year 5 or 6 or the person responsible for health and well-being in the school. The interviews will examine perspectives on the operation of the Action 3:30 trial in their school, including the perceived value of training TAs, why the school signed up to the trial, aspects that did and did not work, and suggested refinements to the intervention. Intervention school administrators will also be asked to comment on what resources, training and funding would be necessary to continue the provision of the activity sessions once the trial funding has ended.

### Sample size

The target sample size is 30 Year 5 and 6 children per school, thus yielding 300 intervention and 300 control participants. The feasibility trial is being conducted to provide the information necessary to conduct an adequately powered cluster randomized controlled trial (cluster RCT). Previous research suggests that a cluster RCT that is designed to detect change in accelerometer-determined PA levels could involve between 40 and 80 schools [35,36]. Thus, to estimate the sample size for such a trial there is a need to determine variability in PA and intraclass correlation coefficient. Although we have not conducted a conventional sample size estimate for between-group comparisons, 600 participants will provide a margin of error of ±6% for adherence to intervention and ±4% for collection of outcome data.

### Data and economic analyses

#### Quantitative analyses

As a feasibility trial, the main analyses will focus on data relating to recruitment, intervention adherence and feasibility of data collection, and will mainly be descriptive in nature (means (with standard deviation) or *N* (number, as a percentage), as appropriate). School associated intraclass correlation coefficient s will be estimated for possible primary outcomes for a future trial. Mean session attendance will be calculated. The loss to follow-up rate for both groups will be identified. We will estimate between-group differences in MVPA using appropriate multi-level regression models, but the focus will be on the inclusion or exclusion of meaningful effects within the 95% confidence intervals. *P* values will not be considered.

The economic analyses will estimate the direct and indirect resource use collected in line with the direct cost items listed in recent NICE guidance [37] and associated costs of programme delivery for the students themselves and their parents and carers. A framework for an appropriate, relevant and feasible trial economic evaluation will be developed.

#### Qualitative analysis

All focus group and interview recordings will be transcribed verbatim and anonymized. As the data are exploratory, we will adopt a thematic analytical approach. Meaningful content will be coded and codes grouped to form themes that describe the content of codes [38]. Quotations deemed to best represent the nature of each theme will then be extracted. This process will be conducted independently for the pupil focus groups, TAs and school administrators. Data from the three sources will then be compared to establish if there are any consistencies and discrepancies in the reports from the three groups.

## Discussion

This paper describes the protocol for the Action 3:30 feasibility trial, which is attempting to increase PA among Year 5 and 6 children attending UK primary schools. Many children do not engage in sufficient amounts of PA. Primary school is a key period for the establishment of PA behaviours, preferences and skills. The after-school period is well-established for the delivery of extracurricular programmes but there is an absence of well-researched, well-evaluated after-school interventions. Action 3:30 is a new intervention that is designed to train TAs to deliver PA programmes in UK primary schools. The goal of the feasibility trial is to assess the potential of this innovative intervention approach and provide all the information necessary to design a cluster RCT.

## Trial Status

Intervention on-going with contact sessions due to end July 2013 and data collection due to be completed by December 2013.

### Current study status (11/09/2012)

We have obtained ethical approval and funding for the Action 3:30 study and have recruited all project staff. Invitations to participate in the study were sent to 132 schools (31, Bristol City Council; 51, Bath and North East Somerset Council; 50 South Gloucestershire Council) within a 20-mile radius of the university department that was not participating in current university evaluations of school-based PA interventions. Twenty schools consented to participate (a further three were added to a reserve list), two expressed an interest but did not enrol due to a shortage of Year 5 and 6 pupils and a shortage of space. One hundred and seven schools did not reply. Participant recruitment began in all 20 schools in September 2012 with baseline data collection due to be completed by late October 2012. TA training will commence in mid-November 2012.

## Abbreviations

CDO: Coach Development Officer; CSLA: Community Sports Leader Award; MVPA: moderate-to-vigorous intensity physical activity; NICE: National Institute for Health and Clinical Excellence; PA: physical activity; PE: physical education; RCT: randomized controlled trial; TA: teaching assistant.

## Competing interests

We have no competing interests to declare.

## Authors’ contributions

The study was conceived by RJ, ARC, KRF, AAM, JP, SJS and JLT. MJE is the project coordinator and MS is the project statistician. The first draft of the paper was produced by RJ with input from all other authors. All authors have edited and critically reviewed the paper for intellectual content and approved the final version of the paper.
